# Supplementary material for: Bioinformatics analysis identifies several intrinsically disordered human E3 ubiquitin-protein ligases
Source: PeerJ. 2016 Feb 25;4:e1725. doi: 10.7717/peerj.1725 (PMC4782732; doi:10.7717/peerj.1725)
Supplement: Figure S1 [file peerj-04-1725-s001.pdf]

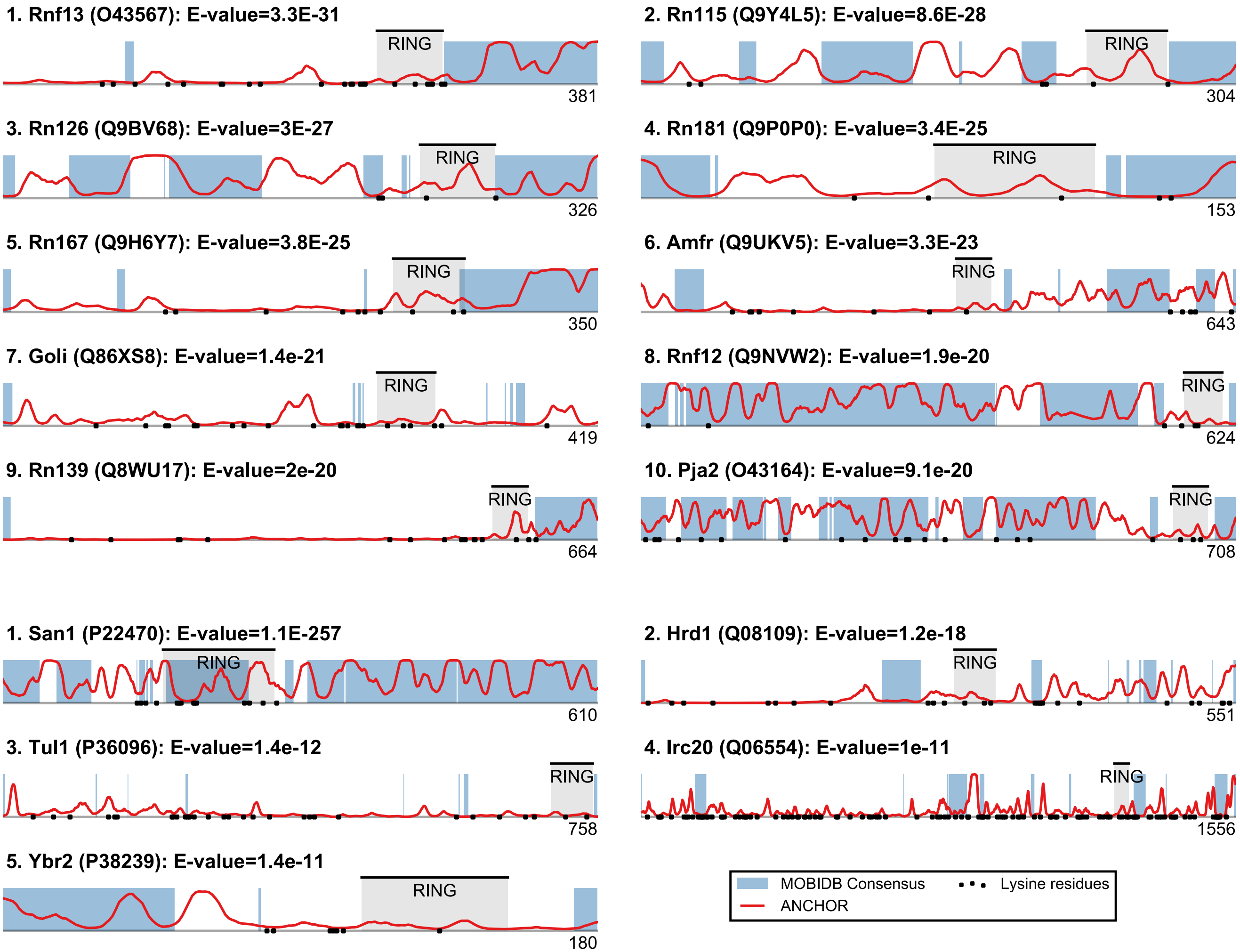
1. Rnf13 (O43567): E-value=3.3E-31
RING
381
2. Rn115 (Q9Y4L5): E-value=8.6E-28
RING
304
3. Rn126 (Q9BV68): E-value=3E-27
RING
326
4. Rn181 (Q9P0P0): E-value=3.4E-25
RING
153
5. Rn167 (Q9H6Y7): E-value=3.8E-25
RING
350
6. Amfr (Q9UKV5): E-value=3.3E-23
RING
643
7. Goli (Q86XS8): E-value=1.4e-21
RING
419
8. Rnf12 (Q9NVW2): E-value=1.9e-20
RING
624
9. Rn139 (Q8WU17): E-value=2e-20
RING
664
10. Pja2 (O43164): E-value=9.1e-20
RING
708
1. San1 (P22470): E-value=1.1E-257
RING
610
2. Hrd1 (Q08109): E-value=1.2e-18
RING
551
3. Tul1 (P36096): E-value=1.4e-12
RING
758
4. Irc20 (Q06554): E-value=1e-11
RING
1556
5. Ybr2 (P38239): E-value=1.4e-11
RING
180
MOBIDB Consensus
Lysine residues
ANCHOR
