# Supplementary figures and images for: Bioinformatics analysis identifies several intrinsically disordered human E3 ubiquitin-protein ligases
Source: PeerJ. 2016 Feb 25;4:e1725. doi: 10.7717/peerj.1725 (PMC4782732; doi:10.7717/peerj.1725)

## Bag6 (P46379)

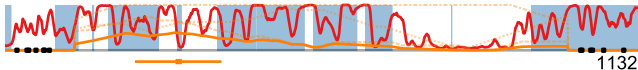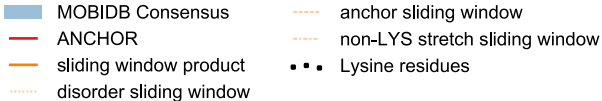

Supplement: Figure S2 [file peerj-04-1725-s002.pdf]
